# Multimodal evoked potentials are useful for the diagnosis of pediatric acute disseminated encephalomyelitis

**DOI:** 10.1186/s12887-024-04576-7

**Published:** 2024-02-02

**Authors:** Jing Liu, Mei Jin, Meijie Zhang, Yonggang Wang, Suzhen Sun

**Affiliations:** 1 The Children Hospital of Hebei Province, Shijiazhuang, Hebei 050000 China; 2 The Key Laboratory of Pediatric Epilepsy and Neurological Disorders of Hebei Province, Shijiazhuang, Hebei 050000 China

**Keywords:** Acute disseminated encephalomyelitis, Children, Brainstem auditory evoked potentials, Visual evoked potential, Somatosensory evoked potential, Early diagnosis

## Abstract

**Background:**

The application of evoked potentials (EPs) to the diagnosis of acute disseminated encephalomyelitis (ADEM ) has not been investigated in detail. The aim of this study, therefore, was to analyze the value of multimodal EPs in the early diagnosis of pediatric ADEM.

**Methods:**

This was a retrospective study in which we enrolled pediatric ADEM patients and controls (Cs) from neurology units between 2017 and 2021. We measured indices in patients using brainstem auditory evoked potentials (BAEPs), visual evoked potentials (VEPs) and somatosensory evoked potentials (SEPs), and then we analyzed their early diagnostic value in ADEM patients.

**Results:**

The mean age of the ADEM group was 6.15 ± 3.28 years (range,1–12 years) and the male/female ratio was 2.1:1 The mean age of the Cs was 5.97 ± 3.40 years (range,1–12 years) and the male/female ratio was 1.3:1. As we used magnetic resonance imaging (MRI) as the diagnostic criterion, the sensitivity, specificity, and accuracy (κ was 0.88) of multimodal EPs were highly consistent with those of MRI; and the validity could be ranked in the following order with respect to the diagnosis of ADEM: multimodal Eps > single SEP > single VEP > single BAEP. Of 34 patients with ADEM, abnormalities in multimodal EPs were 94.12%, while abnormalities in single VEPs, BAEPs and SEPs were 70.59%,64.71%and 85.3%, respectively. We noted significant differences between single VEP/BAEPs and multimodal EPs (χ^2^ = 6.476/8.995,*P* = 0.011/0.003).

**Conclusions:**

The combined application of multimodal EPs was superior to BAEPs, VEPs, or SEPs alone in detecting the existence of central nerve demyelination, and we hypothesize that these modalities will be applicable in the early diagnosis of ADEM.

## Introduction

Multimodal evoked potentials (EPs) include brainstem auditory evoked potentials (BAEPs), visual evoked potentials (VEPs), and somatosensory evoked potentials (SEPs). EPs can indicate lesions in the central nervous system (CNS) in an objective and sensitive manner [1]. BAEPs are recorded in superficial bipolar electrodes (A1or A2, relative to Cz) through the auditory conduction pathway after applying a sound stimulus [2]. A VEP is the evoked response recorded in the visual center after applying a light stimulus, and is the total response of visual signal generation, transduction, and final transmission to the visual center [3]. Furthermore, and an SEP is the evoked response recorded in the corresponding sensory cortex through the deep sensory conduction pathways following electrical stimulation of the limbs [4].

Acute disseminated encephalomyelitis (ADEM) is an immune-mediated idiopathic inflammatory demyelinating disease of the CNS and is characterized by an encephalopathy ranging from behavioral change to alteration in consciousness [5]. Many studies have shown that ADEM is associated with a favorable outcome [6], whereas other studies revealed a mortality rate of 20% and considerable neurologic sequelae in survivors [7, 8]. Therefore, it is critical to identify ADEM through clinical presentations, imaging findings, neurophysiological techniques and immunological testing at the acute stage. Many researchers have investigated multimodal EPs of CNS demyelinating diseases such as multiple sclerosis [9], neuromyelitis optica spectrum disorder [10], and other diseases with clinical features of CNS damage; the latter include chronic HCV infection [11], and primary Sjögren’s Syndrome [12]. However, relatively few studies on multimodal EPs have been conducted on ADEM, particularly in children. Thus, we herein comprehensively investigated the diagnostic value of multimodal EPs in ADEM from auditory, visual, and sensory aspects and thereby retrospectively analyzed the BAEP, VEP, and SEP characteristics of 34 children with ADEM.

## Materials and methods

### Subjects

The clinical data from children visiting the Neurology Department of our hospital between November 2017 and December 2021 were analysed retrospectively. Inclusion criteria for the ADEM group were ① age ≥ 1 and ≤ 16 years; ② fulfillment of the diagnostic criteria of ADEM based on the International Pediatric MS Study Group (IPMSSG) recommendations [13], in which an acute onset of neurologic disturbance with polysymptomatic presentation and brain MRI changes involving the white matter in the distribution manifest a diffuse disseminated demyelinating disease; and ③ availability of complete and well-documented case records. Exclusion criteria were ① a history of demyelinating disease or other underlying neurological disease before ADEM onset, ②follow-up examination consistent with multiple sclerosis, neuromyelitis optica, or systemic lupus erythematosus, ③connective tissue diseases and tumors complicated by demyelinating diseases of the CNS or inborn errors of metabolism, and ④ lack of consent to follow-up examination. Inclusion criteria for the control group were ① age ≥ 1 and ≤ 16 years ; ② diagnosis with psychiatric disorders, migraine and benign intracranial hypertension, and no clinical features of CNS damage as well as any other autoimmune, or oncological diseases; ③ normal brain magnetic resonance imaging (MRI), and ④ a normal white blood cell count or cerebrospinal fluid (CSF) protein.This study was approved by the Ethics Committee of the Children’s Hospital of Hebei Province.

### Clinical data and testing methods

#### Clinical and laboratory data

We analyzed the clinical features of age, sex, prior infection, clinical manifestations, cranial nerve damage, sensory impairment, autonomic dysfunction, therapeutic drugs, and CSF protein concentration and white cell counts (pleocytosis was defined as white cell counts of more than 10 × 10^6^/L; increased CSF protein concentration was defined as over 0.4 g·L^− 1^).

#### MR imaging protocols

Imaging was performed on a 3.0T Signa Hdx MR unit with a head 16-channel coil. The first plain MR sequences were generated as follows: axial, sagittal, and coronal T2-weighted images, axial T1-weighted and fluid-attenuated inversion recovery sequence images. All neuroimaging was reviewed by a neuroradiologist (C.S.) who was unaware of the patient’s identity,clinical presentation, or original radiology report. Brain MRIs with large (> 2 cm axial), hazy, and bilateral lesions were defined as typically MRI-positive.

#### Evoked potential protocols

The procedures for EPs were conducted according to the International Federation of Clinical Neurophysiology guidelines [14, 15]. ① For BAEP measurements, we performed the examination in a soundproof room using disk-shaped silver chloride electrodes. BAEP clicks were recorded in the A1/Cz and A2/Cz electrodes. The stimulus frequency was 10.7 Hz, the stimulus intensity was 90 dBnHL and the number of sweeps was 1000. The test was repeated ≥ two or three times per ear. and the mean peak latencies of waves I, III, and V were used as primary observational indicators. ② VEP measurements were performed in a dark room, and VEPs to black and white pattern-reversal stimuli or flash stimuli were recorded at the Oz electrode with reference to the Fz electrode. The pattern-reversal VEP was conducted with a chessboard pattern of black and white squares that were aired on a TV monitor from a distance of 1 m. There were stimuli to both the left and right eye at a frequency of 1Hz. A flash VEP was provided monocularly with intermittent red-diode light stimulation that emanated from goggles placed over the eyes. The average number was 100. The stimulation was repeated three times or more per eye and the P100/P1 latency was used as the primary observational indicator. ③ For SEP measurements, the median nerve SEPs of both upper extremities (UEs) were recorded with surface disk-shaped electrodes placed at C3’/C4’ (2 cm posterior to C3/C4), the fifth spinous process, and Erb’s (Erb) points. The reference electrode was placed at Fz. The tibial nerves SEPs of both lower extremities (LEs) were recorded at Cz’ (2 cm posterior to Cz), spinous process of the first lumbar vertebra and the ipsilateral popliteal fossa, the reference electrodes were placed at contralateral C3 or C4, contralateral iliac crest and adjacent knee bone, respectively. The median nerve was stimulated at the wrist with a repetition rate of 5 Hz, and the tibial nerve was stimulated at the ankle with a repetition rate of 2 Hz. The stimulus intensity was above motor threshold and the stimulation of duration was 0.2 msec. The wave N9/N13/N20/P25 latencies of the upper limbs and the wave N21/P40 latencies of the lower limbs were used as the primary observational indicators. Results outside the 97.5 percentiles of values obtained in the age- and sex-matched Cs as well as the absence of waveform were considered anomalies.

### Statistical analyses

Categorical data are depicted as proportions, and continuous data are shown as the mean ± SD or medians with IQR; and we applied the SPSS statistical package (SPSS, Inc., version 24, Chicago, IL, USA). Differences in proportions were tested by χ^2^ tests or Fisher’s exact test. Independent saµples were evaluated with the *t* test or Wilcoxon rank-suµ test. The sensitivity, specificity, positive and negative predictive value, accuracy, and kappa value (κ value) of single and combined applications of BAEPs, VEPs, and SEPs were calculated. A κ value between 0.4 and 0.75 was considered to be moderately consistent, ≥ 0.75 was highly consistent, and ≤ 0.40 indicated poor consistency. Statistical significance was set at 0.05.

## Results

### Baseline clinical characteristics

**Fig. 1:**
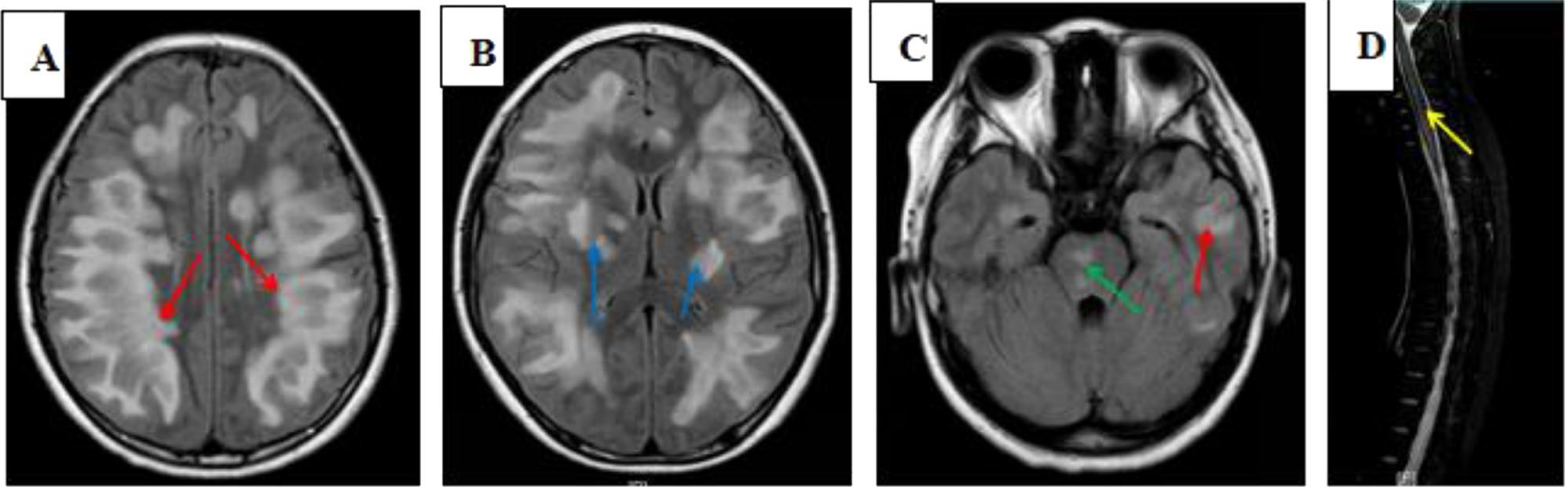
Typical brain and spinal T2-weighted MRI were recorded in 3-year-old patient with ADEM: widespread, blurred (arrowhead), large lesions involving both hemispheres (**A**), the thalamus and brainstem (**B** and **C**), and longitudinally extensive myelitis (**D**)

There were 34 children in the ADEM group, comprising 23 boys and 11 girls, with a mean age of 6.15 ± 3.28 years; the control group was composed of 30 children, 17 boys and 13 girls, with a mean age of 5.97 ± 3.40 years. There was no significant difference in sex or age between the Cs and the ADEM group at the initial medical examination. After disease onset, all children were treated with methyl-prednisolone combined with an intravenous globulin and all underwent cranial and spinal imaging (Fig. 1). Other clinical features of ADEM were summarized in Table 1.

**Fig. 2:**
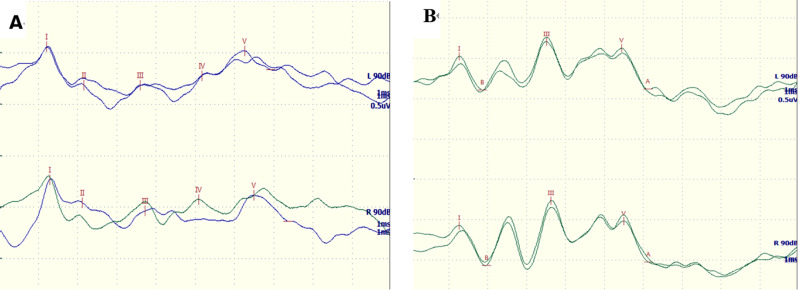
Abnormal BAEPs were recorded in 3-year-old patient with ADEM, prolonged latencies: III (L-3.68 ms, R-3.79 ms) and V (L-6.32 ms, R-6.49 ms)(**A**). The normal BAEPs were elicited from a 4-year-old boy, normal latencies: III (L-3.39 ms, R-3.49 ms) and V (L-5.39 ms, R-5.45 ms)(**B**)

**Fig. 3:**
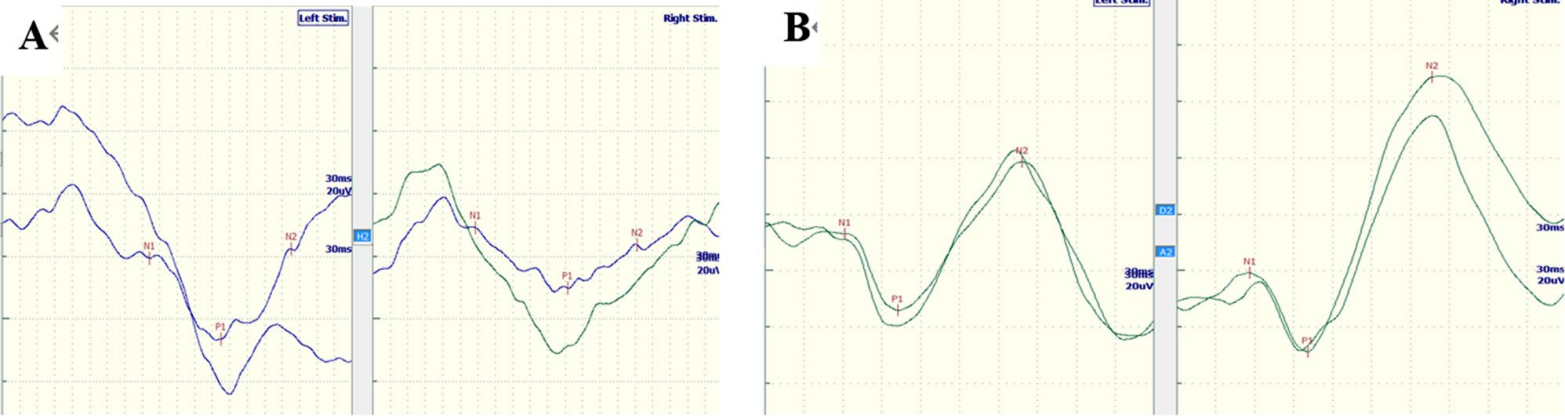
Abnormal VEPs were recorded in 3-year-old patient with ADEM, P1 latencies was L-196 ms and R-204 ms (**A**). The normal VEPs were elicited from a 4-year-old boy, P1 latencies were L-125 ms and R-129 ms)(**B**)

**Fig. 4:**
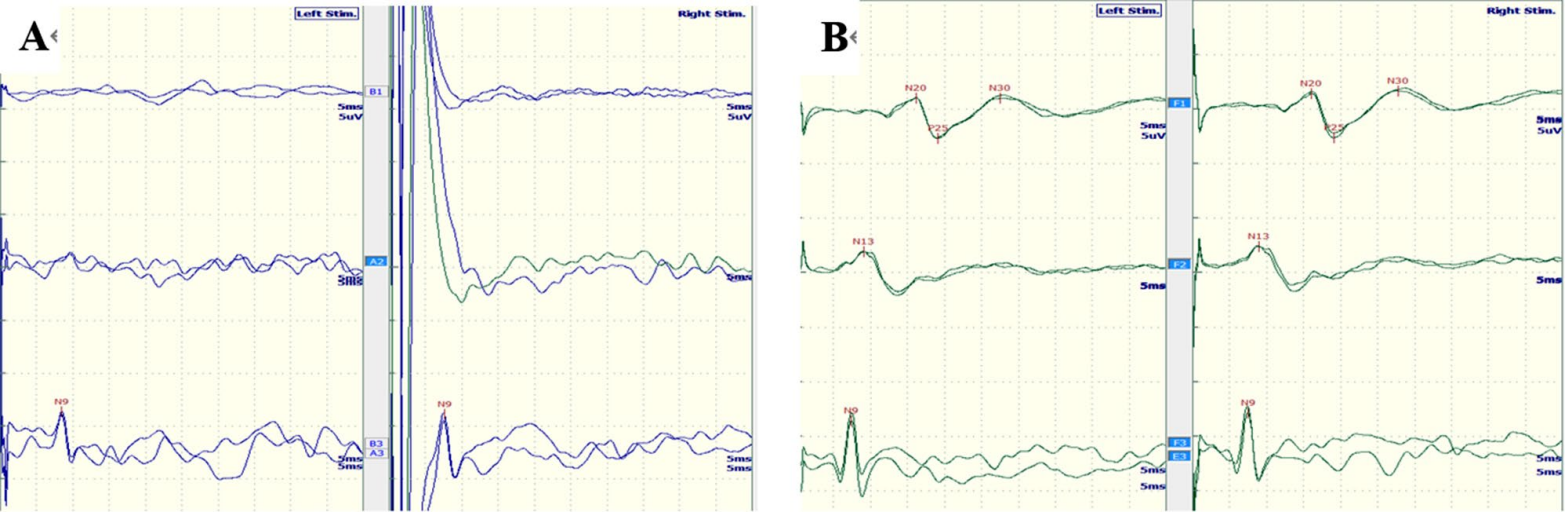
Abnormal SEPs were recorded in 3-year-old patient with ADEM (**A**). The normal SEPs were elicited from a 4-year-old boy, N20 latencies (L-16.2 ms, R-17.1 ms) and P25 latencies (L-19.2 ms, R-19.4ms)(**B**).

When investigating the multimodal EPs, 64.71% (22/34) of the children with ADEM exhibited an abnormal BAEP, including 18 cases with prolonged latency of waves III/V and four cases with unelicited III/V waveforms (Fig. 2; Table 2). In addition, 70.59% (24/34) of children showed abnormal VEPs, comprising nine cases with prolonged P1 latency and 15 cases with prolonged P100 latency (Fig. 3;Table 2). Additionally, 82.4% (28/34) of ADEM children had abnormal UE SEPs, comprising 13 cases with prolonged N20 and P25 latencies and 15 cases with unelicited N20 and P25 waveforms (Fig. 4; Table 2); and 85.3% (29/34) of ADEM children had abnormal LE SEPs, comprising 13 cases with prolonged P40 latencies and 16 cases with unelicited P40 waveforms.

### Application of single EPs and multimodal EPs in the diagnosis of ADEM

As MRI constituted the diagnostic criteria, the sensitivity, specificity, and accuracy (κ was 0.88) of multimodal EPs were highly consistent with those of MRI; and the validity was ranked in the following order with respect to the diagnosis of ADEM: multimodal EPs > single SEP > single VEP > single BAEP (Table 3).

We observed abnormalities in multimodal EPs in 94.12% of the 34 patients with ADEM: these were in single VEPs, BAEPs, and SEPs at proportions of 70.59%, 64.71%, and 85.3%, respectively. While there were significant differences between the single VEPs/BAEPs and multimodal EPs (χ^2^= 6.476/8.995, *P* = 0.011/0.003), there were no differences between the single SEPs and multimodal EPs (*P* = 0.427).

**Table 1:** Clinical characteristics between ADEM patients and controls

Variables	ADEM patients	Cs	Statistic values	P- value
Numbers	34	30		
Age at onset,years, mean ± SD	6.15 ± 3.28	5.97 ± 3.40	*t* = 0.216	0.830
Male, n (%)	23(67.6)	17(56.7)	*χ*^*2*^=0.820	0.365
Preceding event, n (%)				
Respiratory infection	5(14.7)			
Vaccination	3(8.8)			
None	26(76.5)			
Time from onset to admission,days, mean ± SD	5 ± 0.89			
Neurological symptoms, n (%)				
Fever,n(%)	20(58.8)			
Headache,n(%)	14(41.2)			
Vomiting,n(%)	10(29.4)			
Alteration in behavioral, n(%)	24(70.6)			
Muscle weakness,n(%)	23(67.6)			
Sensory disturbance,n(%)	2(5.9)			
Ataxia,n(%)	3(8.8)			
Facial nerve palsy,n(%)	5(14.7)			
Convulsion,n(%)	9(26.5)			
Alteration in consciousness, n(%)	28(82.4)			
Optic neuritis,n(%)	5(14.7)			
Autonomic dysfunction, n (%)	13(38.2)			
Mechanical ventilation,n(%)	3(8.8)			
ICU hospitalization,days,mean ± SD	16 ± 2.5			
Treatment (methyl-prednisolone + IVIg), n(%)	34(100)			
Abnormality Brain MRI,n(%)	34(100)			
Abnormality Spinal MRI,n(%)	29(85.3)			
Abnormality CSF,n(%)				
Pleocytosis	25(73.5)			
Proteins increased	13(38.2)			
MOG antibodies positive,n(%)	4(11.8)			
Abnormality BAEP ,n(%)	22(64.71)	1(3.3)		
Abnormality VEP,n(%)	24(70.59)	2(6.6)		
Abnormality UE SEP,n(%)	28(82.35)	0		
Abnormality LE SEP,n(%)	29(85.29)	1(3.3)		

*Note* ADEM = Acute disseminated encephalomyelitis; Cs = Controls; MOG antibodies = Myelin oligodendrocyte glycoprotein antibodies; BAEP = Brainstem auditory evoked potential; VEP = Visual evoked potential; UE SEP = Upper extremity somatosensory evoked potential; LE SEP = lower extremity somatosensory evoked potential

**Table 2:** Comparison of multimodal evoked potentials in ADEM patients and controls

Variables	ADEM patients	controls	Statistic values	P-value
BAEP				
Numbers	34	30		
Wave I, latency,msec, median(IQR)	1.2(1.2–1.3)	1.2(1.18–1.3)	Z = 0.230	0.818
Numbers	30	30		
Wave III, latency,msec, median(IQR)	3.65(3.55–3.83)	3.3(3.2–3.4)	Z = 5.773	< 0.001
Wave V, latency,msec, median(IQR)	5.71(5.47–5.96)	5.05(4.8–5.23)	Z = 6.246	< 0.001
VEP(≤ 4y)				
Numbers	13	12		
P1,latency,msec,median(IQR)	144.7(109.5-169.7)	129(120.8-135.8)	Z = 1.742	0.082
VEP(>4y)				
Numbers	21	18		
P100,latency,msec,median(IQR)	144.7(126.5-171.6)	105(101.5-110.5)	Z = 4.606	< 0.001
UE SEP				
Numbers	19	30		
N20,latency,msec, median(IQR)	19.8(15.9–21.3)	17(16.1–18.2)	Z = 2.933	0.003
P25,latency,msec, median(IQR)	24.6(20.7–25.8)	19(18.2–21.1)	Z = 3.852	< 0.001
LE SEP				
Numbers	18	30		
P40,latency,msec, median(IQR)	44(37-47.3)	36(34.1–37.4)	Z = 4.510	< 0.001

**Table 3:** The sensitivity, specificity, positive and negative predictive values, accuracy of single EP and multimodal EPs application in children with ADEM

Variables	sensitivity	specificity	PPV	NPV	accuracy	kappa
BAEP + VEP + SEP	94.12	93.33	94.12	93.33	93.75	0.88 ± 0.06
BAEP	64.71	96.67	95.65	70.73	79.69	0.60 ± 0.09
VEP	70.59	93.33	92.31	73.68	81.25	0.63 ± 0.09
UE SEP	82.4	100	100	83.3	90.63	0.81 ± 0.07
LE SEP	85.3	96.7	96.7	85.3	90.63	0.81 ± 0.07

## Discussion

ADEM is a multifocal monophasic inflammatory disease of the CNS that tends to primarily affect children and young adults [16]. In the present study, 23.5% of our patients reflected a history of infection or vaccination, and the proportion of men was higher, but not significant. ADEM manifests a rapid onset and presents with diverse clinical symptoms, with a small subset of children with ADEM experiencing more than one event, making the diagnosis of pediatric ADEM relatively difficult. There is a paucity of biomarkers for the early diagnosis of ADEM other than typical imaging [17]; and as brain and spinal imaging are normal in the early course of the disease, it is essential to seek sensitive and objective neurophysiological markers that can be applied to identify ADEM at the acute stage.

Recording EPs can detect clinical or subclinical lesions in visual, auditory, sensory, and motor pathways; and they are particularly suitable for young children who cannot cooperate fully with the examination. Characteristic findings of BAEPs are primarily manifested as absent or delayed latencies in CNS demyelinating disorders [18],such as MS/NMOSD,or other diseases with clinical features of CNS damage [19]. In addition, prolonged latency and interpeak intervals of waves were expressions of demyelinating damage and are highly accurate in identifying demyelinating diseases [20]. Another finding of BAEPs encompasses amplitudes and morphological abnormalities, which are expressions of axonal damage that is considered the most important factor in determining disability [21]. The BAEPs of children with ADEM in the present study exhibited prolonged latencies of waves III and V, and a significantly higher rate of abnormalities, whereas the latency of wave I did not change significantly. This indicates that the damage to the auditory conduction pathway in children with ADEM was primarily at the level of the superior olivary complex and inferior colliculus, abnormalities observed corresponded to the clinical localization of the lesion, consistent with most studies [17].

In routine practice, VEPs are proven to be useful in the diagnosis of neurological disorders affecting the visual pathways, including optic neuritis [22], multiple sclerosis, Leber’s hereditary optic neuropathy [23], and central nervous system tumors [24].The P100 latency of pattern VEPs indicated myelin damage while flash VEPs indicated axonal pathology; pattern VEPs were also more sensitive than flash VEPs in detecting optic nerve demyelination [25].Abnormal VEP patterns with preserved normal flash VEPs indicated isolated myelin damage, and abnormalities in both pattern VEPs and flash VEPs indicated myelin and axonal damage in the optic nerve [26].Among 34 children with ADEM, there were five patients with optic neuritis, of whom four showed abnormal pattern VEPs and one manifested abnormal flash VEPs; 19 cases showed abnormal subclinical VEPs. These results agree with a previous report showing that the characteristic VEP finding of patients with ADEM was a delay or a loss of P100/P1, indicating that ADEM primarily affected the central portion of the visual conduction pathway.

With respect to SEPs, (which are used to evaluate the function of afferent sensory pathways), the N20 of upper-limb SEPs originates in the primary sensory cortex of the contralateral parietal lobe, while the N9 notch is generated in the brachial plexus and N13 is a synaptic potential generated in the cervical spinal cord where sensory afferents connect with the posterior spinal cord horn [27], Higher abnormal SEPs thus indicated that the afferent sensory conduction pathway in CNS demyelinating diseases, (such as MS and ADEM) primarily involved the central part of the sensory nerve [28].The high sensitivity of SEPs in this study was similar to that of other demyelinating diseases and indicated the poor differentiation of cortical waves and nearly normal latency in peripheral recording sites.

The present results also supported the diagnostic value of BAEPs/VEPs/SEPs in ADEM. In addition, the combined application of multimodal EPs exhibited statistically significant differences compared with BAEPs or VEPs alone; thus, the combination of multimodal EPs can increase the early diagnostic value of children with ADEM.

Our study was also subject to some limitations. First, we only analyzed the latencies of EPS in this study, and did not evaluate wave amplitudes. Second, this was a small retrospective study that only comprised 34 pediatric ADEM patients. In the future, we will also conduct some prospective clinical studies on the prognosis of children with ADEM based on the present study.

In conclusion, we herein analyzed the EP characteristics of pediatric patients with ADEM and demonstrated that the combined application of multimodal EPs was superior to BAEPs, VEPs, or SEPs alone in detecting central nervous system involvement. We recommend that these EPs would be conducted in all patients with ADEM.

## Data Availability

The datasets generated and/or analysed during the current study are not publicly available due to the data protection and privacy of the patients hospitalized at the Children’s Hospital of Hebei province, but are available from the corresponding author on reasonable request.
